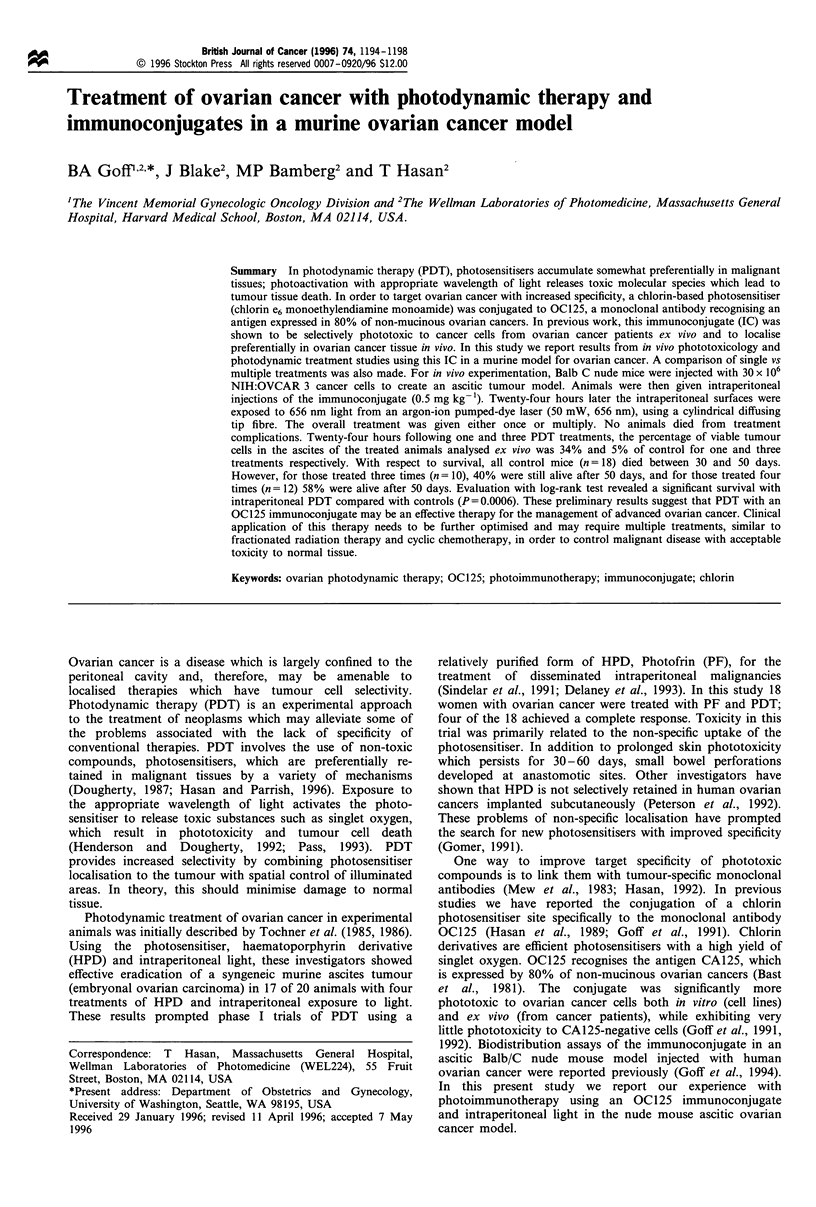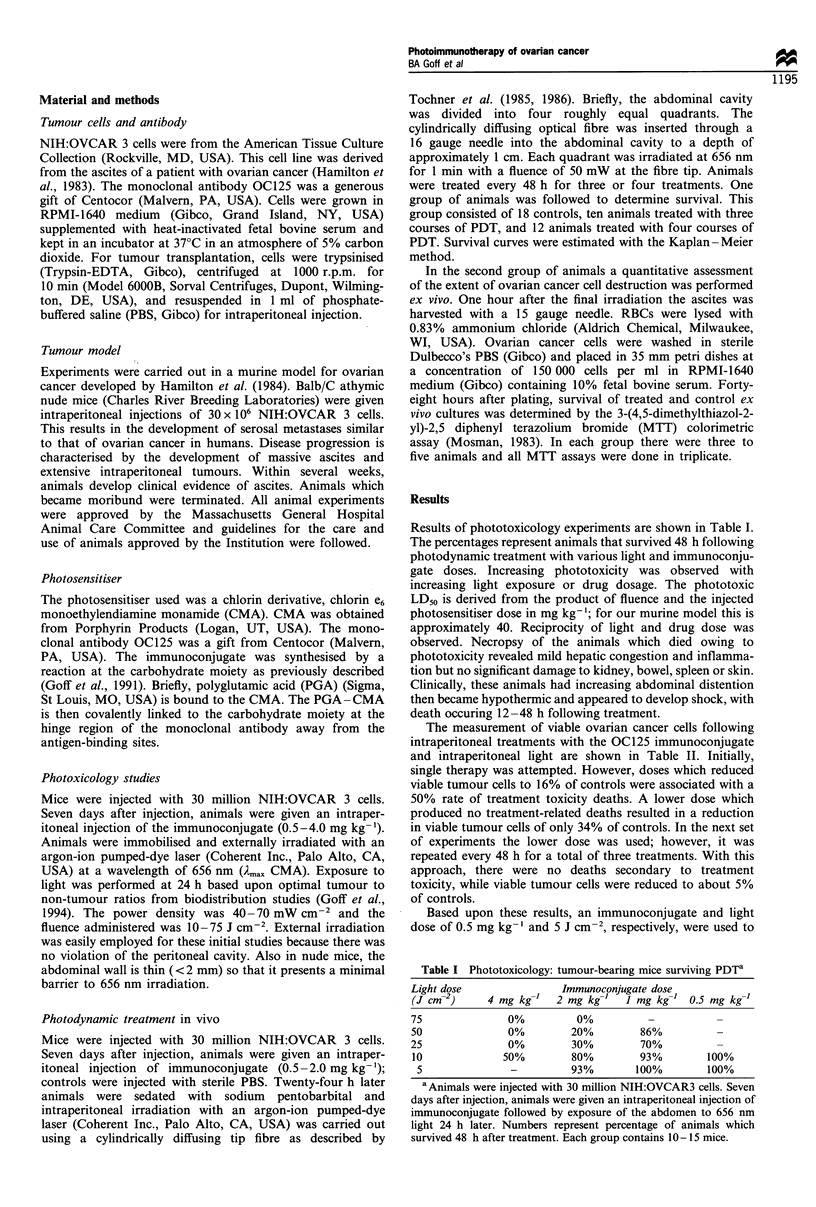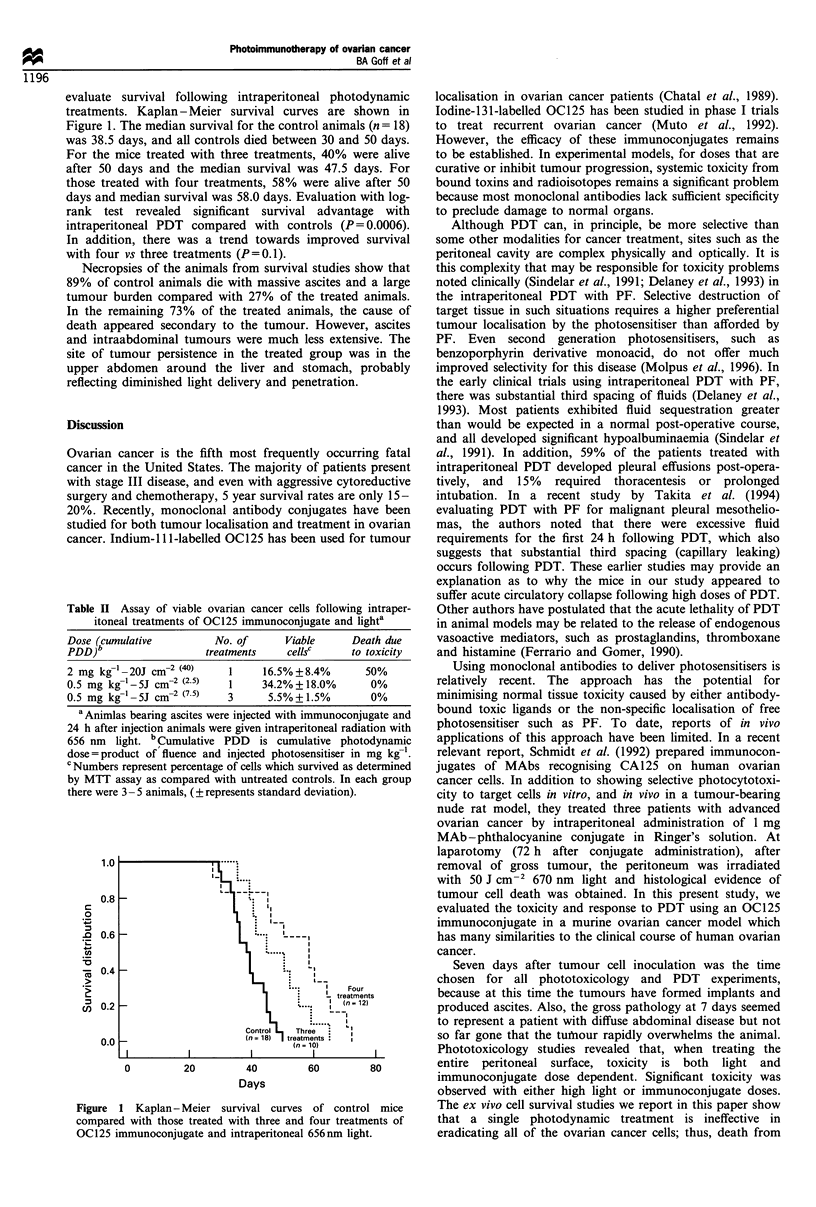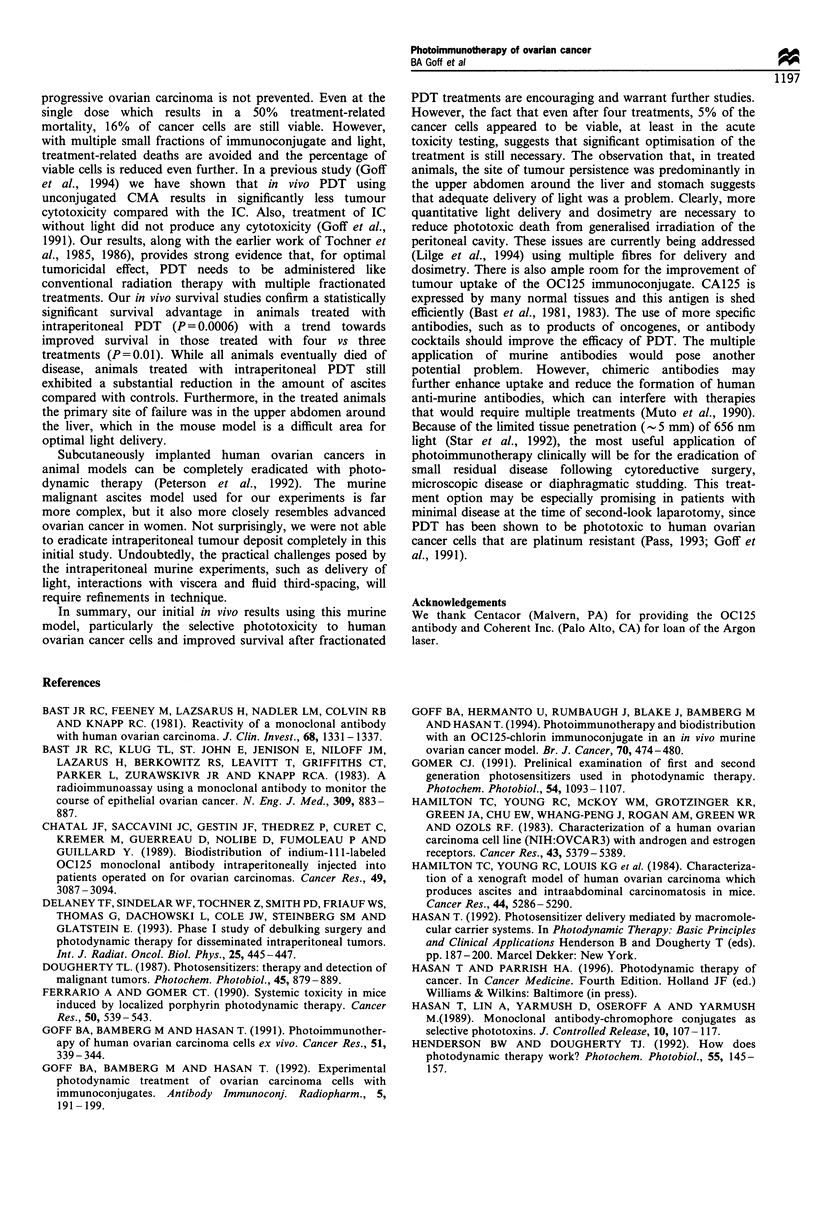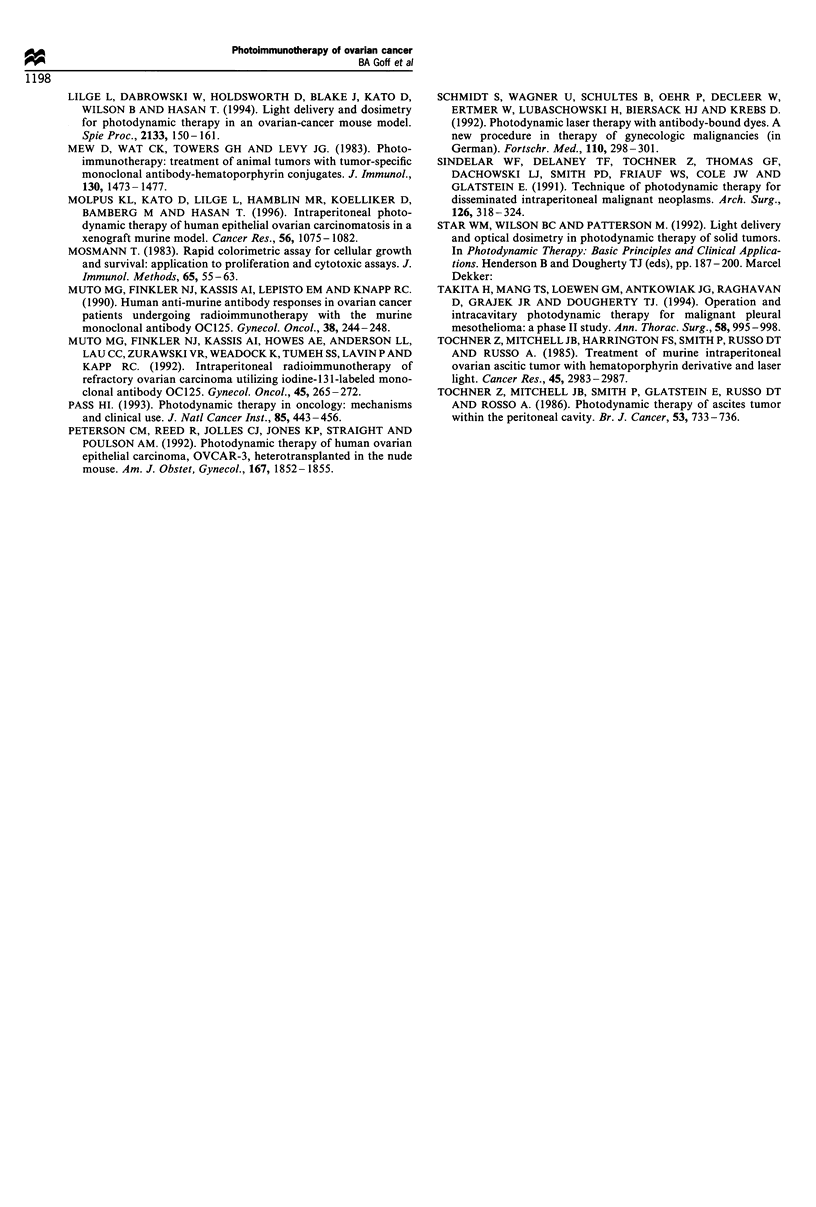# Treatment of ovarian cancer with photodynamic therapy and immunoconjugates in a murine ovarian cancer model.

**DOI:** 10.1038/bjc.1996.516

**Published:** 1996-10

**Authors:** B. A. Goff, J. Blake, M. P. Bamberg, T. Hasan

**Affiliations:** Vincent Memorial Gynecologic Oncology Division, Massachusetts General Hospital, Harvard Medical School, Boston 02114, USA.

## Abstract

In photodynamic therapy (PDT), photosensitisers accumulate somewhat preferentially in malignant tissues; photoactivation with appropriate wavelength of light release toxic molecular species which lead to tumour tissue death. In order to target ovarian cancer with increased specificity, a chlorin-based photosensitiser (chlorin e6 monoethylendiamine monoamide) was conjugated to OC125, a monoclonal antibody recognising an antigen expressed in 80% of non-mucinous ovarian cancers. In previous work, this immunoconjugate (IC) was shown to be selectively phototoxic to cancer cells from ovarian cancer patients ex vivo and to localise preferentially in ovarian cancer tissue in vivo. In this study we report results from in vivo phototoxicology and photodynamic treatment studies using this IC in a murine model for ovarian cancer. A comparison of single vs multiple treatments was also made. For in vivo experimentation, Balb C nude mice were injected with 30 x 10(6) NIH:OVCAR 3 cancer cells to create an ascitic tumour model. Animals were then given intraperitoneal injections of the immunoconjugate (0.5 mg kg-1). Twenty-four hours later the intraperitoneal surfaces were exposed to 656 nm light from an argon-ion pumped-dye laser (50 mW, 656 nm), using a cylindrical diffusing tip fibre. The overall treatment was given either once or multiply. No animals died from treatment complications. Twenty-four hours following one and three PDT treatments, the percentage of viable tumour cells in the ascites of the treated animals analysed ex vivo was 34% and 5% of control for one and three treatments respectively. With respect to survival, all control mice (n = 18) died between 30 and 50 days. However, for those treated three times (n = 10), 40% were still alive after 50 days, and for those treated four times (n = 12) 58% were alive after 50 days. Evaluation with log-rank test revealed a significant survival with intraperitoneal PDT compared with controls (P = 0.0006). These preliminary results suggest that PDT with an OC125 immunoconjugate may be an effective therapy for the management of advanced ovarian cancer. Clinical application of this therapy needs to be further optimised and may require multiple treatments, similar to fractionated radiation therapy and cyclic chemotherapy, in order to control malignant disease with acceptable toxicity to normal tissue.


British Journal of Cancer (1996) 74, 1194-1198
© 1996 Stockton Press All rights reserved 0007-0920/96 $12.00

### Treatment of ovarian cancer with photodynamic therapy and immunoconjugates in a murine ovarian cancer model

BA Goff[1,2,*], J Blake[2], MP Bamberg[2] and T Hasan[2]

[1]*The Vincent Memorial Gynecologic Oncology Division and* [2]*The Wellman Laboratories of Photomedicine, Massachusetts General Hospital, Harvard Medical School, Boston, MA 02114, USA.*

**Summary** In photodynamic therapy (PDT), photosensitisers accumulate somewhat preferentially in malignant tissues; photoactivation with appropriate wavelength of light releases toxic molecular species which lead to tumour tissue death. In order to target ovarian cancer with increased specificity, a chlorin-based photosensitiser (chlorin $e_6$ monoethylendiamine monoamide) was conjugated to OC125, a monoclonal antibody recognising an antigen expressed in 80% of non-mucinous ovarian cancers. In previous work, this immunoconjugate (IC) was shown to be selectively phototoxic to cancer cells from ovarian cancer patients *ex vivo* and to localise preferentially in ovarian cancer tissue *in vivo*. In this study we report results from *in vivo* phototoxicology and photodynamic treatment studies using this IC in a murine model for ovarian cancer. A comparison of single *vs* multiple treatments was also made. For *in vivo* experimentation, Balb C nude mice were injected with $30 \times 10^6$ NIH:OVCAR 3 cancer cells to create an ascitic tumour model. Animals were then given intraperitoneal injections of the immunoconjugate (0.5 mg $kg^{-1}$). Twenty-four hours later the intraperitoneal surfaces were exposed to 656 nm light from an argon-ion pumped-dye laser (50 mW, 656 nm), using a cylindrical diffusing tip fibre. The overall treatment was given either once or multiply. No animals died from treatment complications. Twenty-four hours following one and three PDT treatments, the percentage of viable tumour cells in the ascites of the treated animals analysed *ex vivo* was 34% and 5% of control for one and three treatments respectively. With respect to survival, all control mice ($n=18$) died between 30 and 50 days. However, for those treated three times ($n=10$), 40% were still alive after 50 days, and for those treated four times ($n=12$) 58% were alive after 50 days. Evaluation with log-rank test revealed a significant survival with intraperitoneal PDT compared with controls ($P=0.0006$). These preliminary results suggest that PDT with an OC125 immunoconjugate may be an effective therapy for the management of advanced ovarian cancer. Clinical application of this therapy needs to be further optimised and may require multiple treatments, similar to fractionated radiation therapy and cyclic chemotherapy, in order to control malignant disease with acceptable toxicity to normal tissue.

**Keywords:** ovarian photodynamic therapy; OC125; photoimmunotherapy; immunoconjugate; chlorin

Ovarian cancer is a disease which is largely confined to the peritoneal cavity and, therefore, may be amenable to localised therapies which have tumour cell selectivity. Photodynamic therapy (PDT) is an experimental approach to the treatment of neoplasms which may alleviate some of the problems associated with the lack of specificity of conventional therapies. PDT involves the use of non-toxic compounds, photosensitisers, which are preferentially retained in malignant tissues by a variety of mechanisms (Dougherty, 1987; Hasan and Parrish, 1996). Exposure to the appropriate wavelength of light activates the photosensitiser to release toxic substances such as singlet oxygen, which result in phototoxicity and tumour cell death (Henderson and Dougherty, 1992; Pass, 1993). PDT provides increased selectivity by combining photosensitiser localisation to the tumour with spatial control of illuminated areas. In theory, this should minimise damage to normal tissue.

Photodynamic treatment of ovarian cancer in experimental animals was initially described by Tochner *et al.* (1985, 1986). Using the photosensitiser, haematoporphyrin derivative (HPD) and intraperitoneal light, these investigators showed effective eradication of a syngeneic murine ascites tumour (embryonal ovarian carcinoma) in 17 of 20 animals with four treatments of HPD and intraperitoneal exposure to light. These results prompted phase I trials of PDT using a relatively purified form of HPD, Photofrin (PF), for the treatment of disseminated intraperitoneal malignancies (Sindelar *et al.*, 1991; Delaney *et al.*, 1993). In this study 18 women with ovarian cancer were treated with PF and PDT; four of the 18 achieved a complete response. Toxicity in this trial was primarily related to the non-specific uptake of the photosensitiser. In addition to prolonged skin phototoxicity which persists for 30-60 days, small bowel perforations developed at anastomotic sites. Other investigators have shown that HPD is not selectively retained in human ovarian cancers implanted subcutaneously (Peterson *et al.*, 1992). These problems of non-specific localisation have prompted the search for new photosensitisers with improved specificity (Gomer, 1991).

One way to improve target specificity of phototoxic compounds is to link them with tumour-specific monoclonal antibodies (Mew *et al.*, 1983; Hasan, 1992). In previous studies we have reported the conjugation of a chlorin photosensitiser site specifically to the monoclonal antibody OC125 (Hasan *et al.*, 1989; Goff *et al.*, 1991). Chlorin derivatives are efficient photosensitisers with a high yield of singlet oxygen. OC125 recognises the antigen CA125, which is expressed by 80% of non-mucinous ovarian cancers (Bast *et al.*, 1981). The conjugate was significantly more phototoxic to ovarian cancer cells both *in vitro* (cell lines) and *ex vivo* (from cancer patients), while exhibiting very little phototoxicity to CA125-negative cells (Goff *et al.*, 1991, 1992). Biodistribution assays of the immunoconjugate in an ascitic Balb/C nude mouse model injected with human ovarian cancer were reported previously (Goff *et al.*, 1994). In this present study we report our experience with photoimmunotherapy using an OC125 immunoconjugate and intraperitoneal light in the nude mouse ascitic ovarian cancer model.

Correspondence: T Hasan, Massachusetts General Hospital, Wellman Laboratories of Photomedicine (WEL224), 55 Fruit Street, Boston, MA 02114, USA
*Present address: Department of Obstetrics and Gynecology, University of Washington, Seattle, WA 98195, USA
Received 29 January 1996; revised 11 April 1996; accepted 7 May 1996

### Material and methods

*Tumour cells and antibody*

NIH:OVCAR 3 cells were from the American Tissue Culture Collection (Rockville, MD, USA). This cell line was derived from the ascites of a patient with ovarian cancer (Hamilton *et al.*, 1983). The monoclonal antibody OC125 was a generous gift of Centocor (Malvern, PA, USA). Cells were grown in RPMI-1640 medium (Gibco, Grand Island, NY, USA) supplemented with heat-inactivated fetal bovine serum and kept in an incubator at 37°C in an atmosphere of 5% carbon dioxide. For tumour transplantation, cells were trypsinised (Trypsin-EDTA, Gibco), centrifuged at 1000 r.p.m. for 10 min (Model 6000B, Sorval Centrifuges, Dupont, Wilmington, DE, USA), and resuspended in 1 ml of phosphate-buffered saline (PBS, Gibco) for intraperitoneal injection.

*Tumour model*

Experiments were carried out in a murine model for ovarian cancer developed by Hamilton *et al.* (1984). Balb/C athymic nude mice (Charles River Breeding Laboratories) were given intraperitoneal injections of $30 \times 10^6$ NIH:OVCAR 3 cells. This results in the development of serosal metastases similar to that of ovarian cancer in humans. Disease progression is characterised by the development of massive ascites and extensive intraperitoneal tumours. Within several weeks, animals develop clinical evidence of ascites. Animals which became moribund were terminated. All animal experiments were approved by the Massachusetts General Hospital Animal Care Committee and guidelines for the care and use of animals approved by the Institution were followed.

*Photosensitiser*

The photosensitiser used was a chlorin derivative, chlorin $e_6$ monoethylendiamine monamide (CMA). CMA was obtained from Porphyrin Products (Logan, UT, USA). The monoclonal antibody OC125 was a gift from Centocor (Malvern, PA, USA). The immunoconjugate was synthesised by a reaction at the carbohydrate moiety as previously described (Goff *et al.*, 1991). Briefly, polyglutamic acid (PGA) (Sigma, St Louis, MO, USA) is bound to the CMA. The PGA–CMA is then covalently linked to the carbohydrate moiety at the hinge region of the monoclonal antibody away from the antigen-binding sites.

*Photoxicology studies*

Mice were injected with 30 million NIH:OVCAR 3 cells. Seven days after injection, animals were given an intraperitoneal injection of the immunoconjugate (0.5–4.0 mg $kg^{-1}$). Animals were immobilised and externally irradiated with an argon-ion pumped-dye laser (Coherent Inc., Palo Alto, CA, USA) at a wavelength of 656 nm ($\lambda_{max}$ CMA). Exposure to light was performed at 24 h based upon optimal tumour to non-tumour ratios from biodistribution studies (Goff *et al.*, 1994). The power density was 40–70 mW $cm^{-2}$ and the fluence administered was 10–75 J $cm^{-2}$. External irradiation was easily employed for these initial studies because there was no violation of the peritoneal cavity. Also in nude mice, the abdominal wall is thin (<2 mm) so that it presents a minimal barrier to 656 nm irradiation.

*Photodynamic treatment* in vivo

Mice were injected with 30 million NIH:OVCAR 3 cells. Seven days after injection, animals were given an intraperitoneal injection of immunoconjugate (0.5–2.0 mg $kg^{-1}$); controls were injected with sterile PBS. Twenty-four h later animals were sedated with sodium pentobarbital and intraperitoneal irradiation with an argon-ion pumped-dye laser (Coherent Inc., Palo Alto, CA, USA) was carried out using a cylindrically diffusing tip fibre as described by Tochner *et al.* (1985, 1986). Briefly, the abdominal cavity was divided into four roughly equal quadrants. The cylindrically diffusing optical fibre was inserted through a 16 gauge needle into the abdominal cavity to a depth of approximately 1 cm. Each quadrant was irradiated at 656 nm for 1 min with a fluence of 50 mW at the fibre tip. Animals were treated every 48 h for three or four treatments. One group of animals was followed to determine survival. This group consisted of 18 controls, ten animals treated with three courses of PDT, and 12 animals treated with four courses of PDT. Survival curves were estimated with the Kaplan–Meier method.

In the second group of animals a quantitative assessment of the extent of ovarian cancer cell destruction was performed *ex vivo*. One hour after the final irradiation the ascites was harvested with a 15 gauge needle. RBCs were lysed with 0.83% ammonium chloride (Aldrich Chemical, Milwaukee, WI, USA). Ovarian cancer cells were washed in sterile Dulbecco's PBS (Gibco) and placed in 35 mm petri dishes at a concentration of 150 000 cells per ml in RPMI-1640 medium (Gibco) containing 10% fetal bovine serum. Forty-eight hours after plating, survival of treated and control *ex vivo* cultures was determined by the 3-(4,5-dimethylthiazol-2-yl)-2,5 diphenyl terazolium bromide (MTT) colorimetric assay (Mosman, 1983). In each group there were three to five animals and all MTT assays were done in triplicate.

### Results

Results of phototoxicology experiments are shown in Table I. The percentages represent animals that survived 48 h following photodynamic treatment with various light and immunoconjugate doses. Increasing phototoxicity was observed with increasing light exposure or drug dosage. The phototoxic $LD_{50}$ is derived from the product of fluence and the injected photosensitiser dose in mg $kg^{-1}$; for our murine model this is approximately 40. Reciprocity of light and drug dose was observed. Necropsy of the animals which died owing to phototoxicity revealed mild hepatic congestion and inflammation but no significant damage to kidney, bowel, spleen or skin. Clinically, these animals had increasing abdominal distention then became hypothermic and appeared to develop shock, with death occuring 12–48 h following treatment.

The measurement of viable ovarian cancer cells following intraperitoneal treatments with the OC125 immunoconjugate and intraperitoneal light are shown in Table II. Initially, single therapy was attempted. However, doses which reduced viable tumour cells to 16% of controls were associated with a 50% rate of treatment toxicity deaths. A lower dose which produced no treatment-related deaths resulted in a reduction in viable tumour cells of only 34% of controls. In the next set of experiments the lower dose was used; however, it was repeated every 48 h for a total of three treatments. With this approach, there were no deaths secondary to treatment toxicity, while viable tumour cells were reduced to about 5% of controls.

Based upon these results, an immunoconjugate and light dose of 0.5 mg $kg^{-1}$ and 5 J $cm^{-2}$, respectively, were used to

**Table I** Phototoxicology: tumour-bearing mice surviving PDT[a]

| *Light dose* ($J\ cm^{-2}$) | *Immunoconjugate dose* 4 mg $kg^{-1}$ | 2 mg $kg^{-1}$ | 1 mg $kg^{-1}$ | 0.5 mg $kg^{-1}$ |
|---|---|---|---|---|
| 75 | 0% | 0% | – | – |
| 50 | 0% | 20% | 86% | – |
| 25 | 0% | 30% | 70% | – |
| 10 | 50% | 80% | 93% | 100% |
| 5 | – | 93% | 100% | 100% |

[a] Animals were injected with 30 million NIH:OVCAR3 cells. Seven days after injection, animals were given an intraperitoneal injection of immunoconjugate followed by exposure of the abdomen to 656 nm light 24 h later. Numbers represent percentage of animals which survived 48 h after treatment. Each group contains 10–15 mice.

evaluate survival following intraperitoneal photodynamic treatments. Kaplan–Meier survival curves are shown in Figure 1. The median survival for the control animals ($n=18$) was 38.5 days, and all controls died between 30 and 50 days. For the mice treated with three treatments, 40% were alive after 50 days and the median survival was 47.5 days. For those treated with four treatments, 58% were alive after 50 days and median survival was 58.0 days. Evaluation with log-rank test revealed significant survival advantage with intraperitoneal PDT compared with controls ($P=0.0006$). In addition, there was a trend towards improved survival with four *vs* three treatments ($P=0.1$).

Necropsies of the animals from survival studies show that 89% of control animals die with massive ascites and a large tumour burden compared with 27% of the treated animals. In the remaining 73% of the treated animals, the cause of death appeared secondary to the tumour. However, ascites and intraabdominal tumours were much less extensive. The site of tumour persistence in the treated group was in the upper abdomen around the liver and stomach, probably reflecting diminished light delivery and penetration.

### Discussion

Ovarian cancer is the fifth most frequently occurring fatal cancer in the United States. The majority of patients present with stage III disease, and even with aggressive cytoreductive surgery and chemotherapy, 5 year survival rates are only 15–20%. Recently, monoclonal antibody conjugates have been studied for both tumour localisation and treatment in ovarian cancer. Indium-111-labelled OC125 has been used for tumour localisation in ovarian cancer patients (Chatal *et al*., 1989). Iodine-131-labelled OC125 has been studied in phase I trials to treat recurrent ovarian cancer (Muto *et al*., 1992). However, the efficacy of these immunoconjugates remains to be established. In experimental models, for doses that are curative or inhibit tumour progression, systemic toxicity from bound toxins and radioisotopes remains a significant problem because most monoclonal antibodies lack sufficient specificity to preclude damage to normal organs.

Although PDT can, in principle, be more selective than some other modalities for cancer treatment, sites such as the peritoneal cavity are complex physically and optically. It is this complexity that may be responsible for toxicity problems noted clinically (Sindelar *et al*., 1991; Delaney *et al*., 1993) in the intraperitoneal PDT with PF. Selective destruction of target tissue in such situations requires a higher preferential tumour localisation by the photosensitiser than afforded by PF. Even second generation photosensitisers, such as benzoporphyrin derivative monoacid, do not offer much improved selectivity for this disease (Molpus *et al*., 1996). In the early clinical trials using intraperitoneal PDT with PF, there was substantial third spacing of fluids (Delaney *et al*., 1993). Most patients exhibited fluid sequestration greater than would be expected in a normal post-operative course, and all developed significant hypoalbuminaemia (Sindelar *et al*., 1991). In addition, 59% of the patients treated with intraperitoneal PDT developed pleural effusions post-operatively, and 15% required thoracentesis or prolonged intubation. In a recent study by Takita *et al*. (1994) evaluating PDT with PF for malignant pleural mesotheliomas, the authors noted that there were excessive fluid requirements for the first 24 h following PDT, which also suggests that substantial third spacing (capillary leaking) occurs following PDT. These earlier studies may provide an explanation as to why the mice in our study appeared to suffer acute circulatory collapse following high doses of PDT. Other authors have postulated that the acute lethality of PDT in animal models may be related to the release of endogenous vasoactive mediators, such as prostaglandins, thromboxane and histamine (Ferrario and Gomer, 1990).

Using monoclonal antibodies to deliver photosensitisers is relatively recent. The approach has the potential for minimising normal tissue toxicity caused by either antibody-bound toxic ligands or the non-specific localisation of free photosensitiser such as PF. To date, reports of *in vivo* applications of this approach have been limited. In a recent relevant report, Schmidt *et al*. (1992) prepared immunoconjugates of MAbs recognising CA125 on human ovarian cancer cells. In addition to showing selective photocytotoxicity to target cells *in vitro*, and *in vivo* in a tumour-bearing nude rat model, they treated three patients with advanced ovarian cancer by intraperitoneal administration of 1 mg MAb–phthalocyanine conjugate in Ringer's solution. At laparotomy (72 h after conjugate administration), after removal of gross tumour, the peritoneum was irradiated with 50 J $cm^{-2}$ 670 nm light and histological evidence of tumour cell death was obtained. In this present study, we evaluated the toxicity and response to PDT using an OC125 immunoconjugate in a murine ovarian cancer model which has many similarities to the clinical course of human ovarian cancer.

Seven days after tumour cell inoculation was the time chosen for all phototoxicology and PDT experiments, because at this time the tumours have formed implants and produced ascites. Also, the gross pathology at 7 days seemed to represent a patient with diffuse abdominal disease but not so far gone that the tumour rapidly overwhelms the animal. Phototoxicology studies revealed that, when treating the entire peritoneal surface, toxicity is both light and immunoconjugate dose dependent. Significant toxicity was observed with either high light or immunoconjugate doses. The *ex vivo* cell survival studies we report in this paper show that a single photodynamic treatment is ineffective in eradicating all of the ovarian cancer cells; thus, death from

**Table II** Assay of viable ovarian cancer cells following intraperitoneal treatments of OC125 immunoconjugate and light[a]

| *Dose (cumulative PDD)*[b] | *No. of treatments* | *Viable cells*[c] | *Death due to toxicity* |
|---|---|---|---|
| 2 mg $kg^{-1}$–20J $cm^{-2}$ (40) | 1 | 16.5% ± 8.4% | 50% |
| 0.5 mg $kg^{-1}$–5J $cm^{-2}$ (2.5) | 1 | 34.2% ± 18.0% | 0% |
| 0.5 mg $kg^{-1}$–5J $cm^{-2}$ (7.5) | 3 | 5.5% ± 1.5% | 0% |

[a] Animlas bearing ascites were injected with immunoconjugate and 24 h after injection animals were given intraperitoneal radiation with 656 nm light. [b] Cumulative PDD is cumulative photodynamic dose = product of fluence and injected photosensitiser in mg $kg^{-1}$. [c] Numbers represent percentage of cells which survived as determined by MTT assay as compared with untreated controls. In each group there were 3–5 animals, (± represents standard deviation).

**Figure 1** Kaplan–Meier survival curves of control mice compared with those treated with three and four treatments of OC125 immunoconjugate and intraperitoneal 656 nm light.

progressive ovarian carcinoma is not prevented. Even at the single dose which results in a 50% treatment-related mortality, 16% of cancer cells are still viable. However, with multiple small fractions of immunoconjugate and light, treatment-related deaths are avoided and the percentage of viable cells is reduced even further. In a previous study (Goff *et al.*, 1994) we have shown that *in vivo* PDT using unconjugated CMA results in significantly less tumour cytotoxicity compared with the IC. Also, treatment of IC without light did not produce any cytotoxicity (Goff *et al.*, 1991). Our results, along with the earlier work of Tochner *et al.*, 1985, 1986), provides strong evidence that, for optimal tumoricidal effect, PDT needs to be administered like conventional radiation therapy with multiple fractionated treatments. Our *in vivo* survival studies confirm a statistically significant survival advantage in animals treated with intraperitoneal PDT ($P = 0.0006$) with a trend towards improved survival in those treated with four *vs* three treatments ($P = 0.01$). While all animals eventually died of disease, animals treated with intraperitoneal PDT still exhibited a substantial reduction in the amount of ascites compared with controls. Furthermore, in the treated animals the primary site of failure was in the upper abdomen around the liver, which in the mouse model is a difficult area for optimal light delivery.

Subcutaneously implanted human ovarian cancers in animal models can be completely eradicated with photodynamic therapy (Peterson *et al.*, 1992). The murine malignant ascites model used for our experiments is far more complex, but it also more closely resembles advanced ovarian cancer in women. Not surprisingly, we were not able to eradicate intraperitoneal tumour deposit completely in this initial study. Undoubtedly, the practical challenges posed by the intraperitoneal murine experiments, such as delivery of light, interactions with viscera and fluid third-spacing, will require refinements in technique.

In summary, our initial *in vivo* results using this murine model, particularly the selective phototoxicity to human ovarian cancer cells and improved survival after fractionated PDT treatments are encouraging and warrant further studies. However, the fact that even after four treatments, 5% of the cancer cells appeared to be viable, at least in the acute toxicity testing, suggests that significant optimisation of the treatment is still necessary. The observation that, in treated animals, the site of tumour persistence was predominantly in the upper abdomen around the liver and stomach suggests that adequate delivery of light was a problem. Clearly, more quantitative light delivery and dosimetry are necessary to reduce phototoxic death from generalised irradiation of the peritoneal cavity. These issues are currently being addressed (Lilge *et al.*, 1994) using multiple fibres for delivery and dosimetry. There is also ample room for the improvement of tumour uptake of the OC125 immunoconjugate. CA125 is expressed by many normal tissues and this antigen is shed efficiently (Bast *et al.*, 1981, 1983). The use of more specific antibodies, such as to products of oncogenes, or antibody cocktails should improve the efficacy of PDT. The multiple application of murine antibodies would pose another potential problem. However, chimeric antibodies may further enhance uptake and reduce the formation of human anti-murine antibodies, which can interfere with therapies that would require multiple treatments (Muto *et al.*, 1990). Because of the limited tissue penetration ($\sim 5$ mm) of 656 nm light (Star *et al.*, 1992), the most useful application of photoimmunotherapy clinically will be for the eradication of small residual disease following cytoreductive surgery, microscopic disease or diaphragmatic studding. This treatment option may be especially promising in patients with minimal disease at the time of second-look laparotomy, since PDT has been shown to be phototoxic to human ovarian cancer cells that are platinum resistant (Pass, 1993; Goff *et al.*, 1991).

**Acknowledgements**
We thank Centacor (Malvern, PA) for providing the OC125 antibody and Coherent Inc. (Palo Alto, CA) for loan of the Argon laser.

### References

BAST JR RC, FEENEY M, LAZSARUS H, NADLER LM, COLVIN RB AND KNAPP RC. (1981). Reactivity of a monoclonal antibody with human ovarian carcinoma. *J. Clin. Invest.*, **68**, 1331–1337.

BAST JR RC, KLUG TL, ST. JOHN E, JENISON E, NILOFF JM, LAZARUS H, BERKOWITZ RS, LEAVITT T, GRIFFITHS CT, PARKER L, ZURAWSKIVR JR AND KNAPP RCA. (1983). A radioimmunoassay using a monoclonal antibody to monitor the course of epithelial ovarian cancer. *N. Eng. J. Med.*, **309**, 883–887.

CHATAL JF, SACCAVINI JC, GESTIN JF, THEDREZ P, CURET C, KREMER M, GUERREAU D, NOLIBE D, FUMOLEAU P AND GUILLARD Y. (1989). Biodistribution of indium-111-labeled OC125 monoclonal antibody intraperitoneally injected into patients operated on for ovarian carcinomas. *Cancer Res.*, **49**, 3087–3094.

DELANEY TF, SINDELAR WF, TOCHNER Z, SMITH PD, FRIAUF WS, THOMAS G, DACHOWSKI L, COLE JW, STEINBERG SM AND GLATSTEIN E. (1993). Phase I study of debulking surgery and photodynamic therapy for disseminated intraperitoneal tumors. *Int. J. Radiat. Oncol. Biol. Phys.*, **25**, 445–447.

DOUGHERTY TL. (1987). Photosensitizers: therapy and detection of malignant tumors. *Photochem. Photobiol.*, **45**, 879–889.

FERRARIO A AND GOMER CT. (1990). Systemic toxicity in mice induced by localized porphyrin photodynamic therapy. *Cancer Res.*, **50**, 539–543.

GOFF BA, BAMBERG M AND HASAN T. (1991). Photoimmunotherapy of human ovarian carcinoma cells *ex vivo*. *Cancer Res.*, **51**, 339–344.

GOFF BA, BAMBERG M AND HASAN T. (1992). Experimental photodynamic treatment of ovarian carcinoma cells with immunoconjugates. *Antibody Immunoconj. Radiopharm.*, **5**, 191–199.

GOFF BA, HERMANTO U, RUMBAUGH J, BLAKE J, BAMBERG M AND HASAN T. (1994). Photoimmunotherapy and biodistribution with an OC125-chlorin immunoconjugate in an *in vivo* murine ovarian cancer model. *Br. J. Cancer*, **70**, 474–480.

GOMER CJ. (1991). Prelinical examination of first and second generation photosensitizers used in photodynamic therapy. *Photochem. Photobiol.*, **54**, 1093–1107.

HAMILTON TC, YOUNG RC, MCKOY WM, GROTZINGER KR, GREEN JA, CHU EW, WHANG-PENG J, ROGAN AM, GREEN WR AND OZOLS RF. (1983). Characterization of a human ovarian carcinoma cell line (NIH:OVCAR3) with androgen and estrogen receptors. *Cancer Res.*, **43**, 5379–5389.

HAMILTON TC, YOUNG RC, LOUIS KG *et al.* (1984). Characterization of a xenograft model of human ovarian carcinoma which produces ascites and intraabdominal carcinomatosis in mice. *Cancer Res.*, **44**, 5286–5290.

HASAN T. (1992). Photosensitizer delivery mediated by macromolecular carrier systems. In *Photodynamic Therapy: Basic Principles and Clinical Applications* Henderson B and Dougherty T (eds). pp. 187–200. Marcel Dekker: New York.

HASAN T AND PARRISH HA. (1996). Photodynamic therapy of cancer. In *Cancer Medicine*. Fourth Edition. Holland JF (ed.) Williams & Wilkins: Baltimore (in press).

HASAN T, LIN A, YARMUSH D, OSEROFF A AND YARMUSH M.(1989). Monoclonal antibody-chromophore conjugates as selective phototoxins. *J. Controlled Release*, **10**, 107–117.

HENDERSON BW AND DOUGHERTY TJ. (1992). How does photodynamic therapy work? *Photochem. Photobiol.*, **55**, 145–157.

LILGE L, DABROWSKI W, HOLDSWORTH D, BLAKE J, KATO D, WILSON B AND HASAN T. (1994). Light delivery and dosimetry for photodynamic therapy in an ovarian-cancer mouse model. *Spie Proc.*, **2133,** 150–161.

MEW D, WAT CK, TOWERS GH AND LEVY JG. (1983). Photoimmunotherapy: treatment of animal tumors with tumor-specific monoclonal antibody-hematoporphyrin conjugates. *J. Immunol.*, **130,** 1473–1477.

MOLPUS KL, KATO D, LILGE L, HAMBLIN MR, KOELLIKER D, BAMBERG M AND HASAN T. (1996). Intraperitoneal photodynamic therapy of human epithelial ovarian carcinomatosis in a xenograft murine model. *Cancer Res.*, **56,** 1075–1082.

MOSMANN T. (1983). Rapid colorimetric assay for cellular growth and survival: application to proliferation and cytotoxic assays. *J. Immunol. Methods*, **65,** 55–63.

MUTO MG, FINKLER NJ, KASSIS AI, LEPISTO EM AND KNAPP RC. (1990). Human anti-murine antibody responses in ovarian cancer patients undergoing radioimmunotherapy with the murine monoclonal antibody OC125. *Gynecol. Oncol.*, **38,** 244–248.

MUTO MG, FINKLER NJ, KASSIS AI, HOWES AE, ANDERSON LL, LAU CC, ZURAWSKI VR, WEADOCK K, TUMEH SS, LAVIN P AND KAPP RC. (1992). Intraperitoneal radioimmunotherapy of refractory ovarian carcinoma utilizing iodine-131-labeled monoclonal antibody OC125. *Gynecol. Oncol.*, **45,** 265–272.

PASS HI. (1993). Photodynamic therapy in oncology: mechanisms and clinical use. *J. Natl Cancer Inst.*, **85,** 443–456.

PETERSON CM, REED R, JOLLES CJ, JONES KP, STRAIGHT AND POULSON AM. (1992). Photodynamic therapy of human ovarian epithelial carcinoma, OVCAR-3, heterotransplanted in the nude mouse. *Am. J. Obstet, Gynecol.*, **167,** 1852–1855.

SCHMIDT S, WAGNER U, SCHULTES B, OEHR P, DECLEER W, ERTMER W, LUBASCHOWSKI H, BIERSACK HJ AND KREBS D. (1992). Photodynamic laser therapy with antibody-bound dyes. A new procedure in therapy of gynecologic malignancies (in German). *Fortschr. Med.*, **110,** 298–301.

SINDELAR WF, DELANEY TF, TOCHNER Z, THOMAS GF, DACHOWSKI LJ, SMITH PD, FRIAUF WS, COLE JW AND GLATSTEIN E. (1991). Technique of photodynamic therapy for disseminated intraperitoneal malignant neoplasms. *Arch. Surg.*, **126,** 318–324.

STAR WM, WILSON BC AND PATTERSON M. (1992). Light delivery and optical dosimetry in photodynamic therapy of solid tumors. In *Photodynamic Therapy: Basic Principles and Clinical Applications*. Henderson B and Dougherty TJ (eds), pp. 187–200. Marcel Dekker:

TAKITA H, MANG TS, LOEWEN GM, ANTKOWIAK JG, RAGHAVAN D, GRAJEK JR AND DOUGHERTY TJ. (1994). Operation and intracavitary photodynamic therapy for malignant pleural mesothelioma: a phase II study. *Ann. Thorac. Surg.*, **58,** 995–998.

TOCHNER Z, MITCHELL JB, HARRINGTON FS, SMITH P, RUSSO DT AND RUSSO A. (1985). Treatment of murine intraperitoneal ovarian ascitic tumor with hematoporphyrin derivative and laser light. *Cancer Res.*, **45,** 2983–2987.

TOCHNER Z, MITCHELL JB, SMITH P, GLATSTEIN E, RUSSO DT AND ROSSO A. (1986). Photodynamic therapy of ascites tumor within the peritoneal cavity. *Br. J. Cancer*, **53,** 733–736.